# A narrative review on the applications of intracavitary contrast-enhanced ultrasonography in pediatric lower genitourinary anomalies

**DOI:** 10.3389/fped.2023.984643

**Published:** 2023-05-19

**Authors:** Jiayu Ren, Ting Ma, Shuyan Huang, Gongquan Chen, Christoph F. Dietrich, Yuexiang Peng, Xinwu Cui

**Affiliations:** ^1^ Department of Medical Ultrasound, Tongji Hospital, Tongji Medical College, Huazhong University of Science and Technology, Wuhan, China; ^2^ Department of Ultrasound, The First People’s Hospital of Huaihua, Huaihua, China; ^3^ Department Allgemeine Innere Medizin, Kliniken Hirslanden Bern, Beau Site, Salem und Permanence, Bern, Switzerland; ^4^ Department of Ultrasound, The Third Hospital of Wuhan, Wuhan, China

**Keywords:** children, intracavitary, ultrasound contrast agent, contrast-enhanced voiding urosonography, contrast-enhanced genitosonography, genitourinary anomaly

## Abstract

**Purpose:**

We mainly aimed to perform a narrative review of clinical applications of the three intracavitary contrast-enhanced ultrasonography (CEUS) including contrast-enhanced voiding urosonography (ceVUS), contrast-enhanced retrograde urethrosonography (ceRUG), and contrast-enhanced genitosonography (ceGS) in pediatric lower genitourinary anomalies.

**Method:**

A literature search in the PubMed and Web of Science databases was conducted up to 1 July 2022 on all studies published in English using the search terms “contrast-enhanced voiding urosonography”, “contrast-enhanced retrograde urethrosonography”, and “contrast-enhanced genitosonography”. Trials were limited to pediatric subjects (ages ≤18 years) with no time restrictions. The inclusion criteria were studies on ceVUS, ceRUG, and ceGS to evaluate pediatric lower genitourinary anomalies. Two independent authors summarized the included articles.

**Results:**

Finally, a total of 48 original articles and 6 case reports or case series were included, of which 50 (93%) were only relevant to ceVUS, 3 (5%) articles involved ceGS, while only one (2%) article involved ceRUG, and 87% of the applications of ceVUS were focused on vesicoureteral reflux (VUR). We also searched 24 related reviews, of which 20 involved ceVUS in diagnosing VUR and 4 involved ceRUG and ceGS for other lower genitourinary anomalies.

**Conclusion:**

Intracavitary CEUS including ceVUS, ceRUG, and ceGS in pediatrics has many advantages over other radiological examinations in diagnosing lower genitourinary anomalies. Although ceVUS is widely used in detecting VUR, ceRUG and ceGS have also become promising techniques for evaluating the urethral pathologies and urogenital sinus.

## Introduction

There is a broad disease spectrum of lower genitourinary tract anomalies in children, among which vesicoureteral reflux (VUR) is the most common urological disorder (1). Due to end-stage renal failure and infertility caused by abnormalities of the lower urinary tract and genital tract anomalies, early diagnosis and treatment are important for the overall outcomes and prognoses of the patients (2). Historically, radiographic voiding cystourethrography (VCUG) and direct or indirect radionuclide cystography (RNC) were mainly used in diagnosing pediatric VUR (3), while VCUG and retrograde urethrography were applied for imaging the urethra (4), and the genitography was established to diagnose the urogenital sinus (5). However, due to the prolonged radiation exposure of the children's gonads with VCUG and genitography, and poor anatomical resolution of RNC, a safe and non-radiologic imaging modality, the intracavitary contrast-enhanced ultrasonography (CEUS), was selected alternatively as a further diagnostic option for the lower urinary and genital tract anomalies (6). Based on the administration of ultrasound contrast agents (UCAs) into the bladder, urethra, or perineal orifice through a catheter, intracavitary CEUS including contrast-enhanced voiding urosonography (ceVUS), contrast-enhanced retrograde urethrosonography (ceRUG), and contrast-enhanced genitosonography (ceGS) has become a rapidly developing imaging examination mainly for detecting and grading VUR as well as for diagnosing varieties of urethral diseases and urogenital sinus malformation (7–9). Another intracavitary CEUS application, hysterosalpingo-contrast sonography, with its utility not yet developed in the pediatric population, is not covered in this article.

In this review, we mainly aim to present a comprehensive review of intracavitary CEUS applications (ceVUS, ceRUG, and ceGS) in pediatric genitourinary anomalies including lower urinary tract anomalies and urogenital sinus malformation, which could be helpful for pediatricians and radiologists to choose the right ultrasonographic imaging method when confronted with these diseases. This review article will be presented in three sections. The first section will introduce the evolution history of the three intracavitary CEUS techniques (ceVUS, ceRUG, and ceGS) in pediatrics. In the second section, we will present the appropriate dosages and safety of ceVUS, ceRUG, and ceGS in pediatrics. Lastly, and most importantly, related pediatric clinical applications of ceVUS, ceRUG, and ceGS in lower genitourinary diseases (e.g., VUR, megaureter, ectopic ureter, ureterocele, bladder diverticulum, posterior urethral valves (PUVs), anterior urethral valves (AUVs), diverticula of the prostatic utricle, stricture of the bulbar urethra, spinning top urethra, and urogenital sinus) that available documents refer to will be described.

## Materials and methods

We searched the PubMed and Web of Science databases for all research published in English up to 1 July 2022, using the search terms “contrast-enhanced voiding urosonography”, “contrast-enhanced retrograde urethrosonography”, and “contrast-enhanced genitosonography”. Trials were limited to pediatric subjects (ages ≤18 years) with no time restrictions. We also included some narrative and systematic reviews to provide our readers with adequate details within the allowed number of references. In addition to the database searching, a hand search was performed, consisting of the reference lists of related articles and reviews and Google scholar search engines.

The articles were reviewed to determine the relevance based on the following criteria: studies that involved pediatric subjects using ceVUS, ceRUG, and ceGS to evaluate pediatric lower genitourinary anomalies including VUR, megaureter, ectopic ureter, ureterocele, bladder diverticulum, PUV, AUV, diverticula of the prostatic utricle, stricture of the bulbar urethra, spinning top urethra, and urogenital sinus. The articles that were beyond this coverage or referred to mixed pediatric and adult subjects or adult subjects were excluded.

All the titles and abstracts for all eligible articles were reviewed by two authors independently. If the abstracts were not relevant, then they were discarded, and the full-text articles were accessed. Further reviewing the full-text papers may lead to deserting some irrelevant documents and finally retaining articles that met the inclusion criteria in this review. When there was any discrepancy in the ultimately included articles, a consensus negotiation was reached to form the final inclusion result between the two authors.

## Results

**Figure 1:**
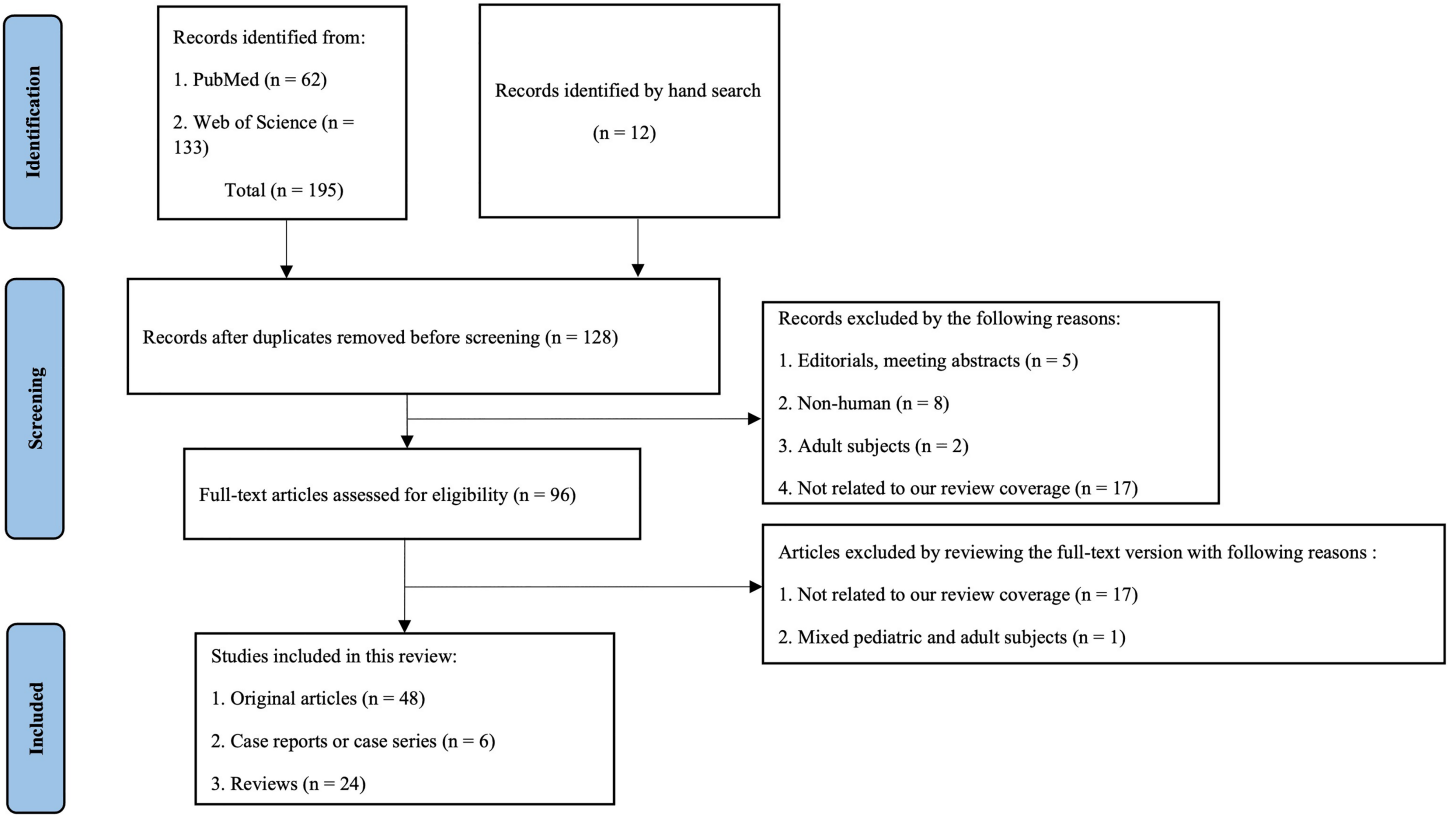
The detailed PRISMA flowchart of the included studies for this review.

Through a comprehensive search in the PubMed and Web of Science databases, a total of 195 records were retrieved for our review up to 1 July 2022, including 62 papers from the PubMed and 133 articles from the Web of Science. After eliminating duplicate literatures preliminarily, two investigators independently reviewed the titles and abstracts of the remaining 128 studies, excluding editorials, meeting abstracts, non-human literature, adult subjects, and not related articles. The 96 full-text papers obtained were further reviewed and eventually included 48 original articles, 6 case reports or case series, and 24 reviews eligible for this review. The detailed PRISMA flowchart is summarized in Figure 1.

Among the 54 included clinical trials, 50 studies (93%) were only related to ceVUS, 3 (5%) articles involved ceGS, while only one (2%) article involved ceRUG. The proportion of published studies involving the use of ceVUS was significantly higher than ceRUG and ceGS, and 87% of the applications of ceVUS were focused on VUR. When referring to the types of UCAs used in these studies, 56% of the studies were implemented with SonoVue®/Lumason®, and 41% of the studies used the first-generation UCAs (Levovist, Echovist, and Albunex) (Table 1).

**Table 1:** Pediatric-only included studies that report the lower urinary anomalies using intracavitary contrast-enhanced ultrasonography including ceVUS, ceRUG, and ceGS performed with UCAs published in English.

Publication year, first author	UCA	Technique	Urinary abnormalities	Side effects
2022, Cvitkovic-Roic (10)	SonoVue®	ceVUS	VUR, IRR	N/A
2022, Seelbach (11)	SonoVue®	ceVUS	VUR, bladder rupture, urogenital malformation	Mild fever, skin rash
2021, Benya (12)	Lumason®	ceVUS	VUR, ureterocele, duplication of the renal collecting system, ureteral duplication	No
2021, Kim (13)	SonoVue®	ceVUS	VUR, IRR	No
2021, Marschner (14)	SonoVue®	ceVUS	VUR	No
2021, Rubelj (15)	SonoVue®	ceVUS	VUR	N/A
2021, Simicic Majce (8)	SonoVue®	ceVUS	VUR, IRR	No
2020, Chow (7)	Optison® Lumason®	ceGS	Urogenital sinus, Cloacal malformation	N/A
2020, Fischer (16)	Optison®	ceVUS	Bulbar urethral stricture	N/A
2020, Rojas-Ticona (17)	N/A	ceVUS	PUV, VUR, anterior urethral mucocele, bladder diverticulum obstructing the urethra	N/A
2020, Siomou (18)	SonoVue®	ceVUS	Primary VUR	No
2020, Yi (19)	N/A	ceVUS	VUR	N/A
2019, Ključevšek (20)	SonoVue®	ceVUS	VUR	No
2019, Velasquez (21)	Lumason®	ceVUS	VUR	N/A
2018, Mane (22)	Second- generation contrast agent	ceVUS	VUR	N/A
2018, Ntoulia (9)	Optison®	ceVUS	VUR	Transient dysuria (1/30)
2018, Patel (23)	Lumason®	ceVUS	Incomplete urethral duplication, posterior bladder diverticulum	N/A
2018, Seranio (24)	Lumason®	ceGS	Urogenital sinus	N/A
2018, Woźniak (25)	SonoVue®	ceVUS	VUR	N/A
2018, Zhang (26)	SonoVue®	ceVUS	VUR	No
2017, Kuzmanovska (27)	SonoVue®	ceVUS	VUR, spinning top urethra	No
2016, Colleran (28)	Optison®	ceVUS	VUR, IRR	N/A
2016, Piskunowicz (29)	SonoVue®	ceVUS	VUR	No
2016, Woźniak (30)	SonoVue®	ceVUS	VUR	N/A
2015, Babu (31)	SonoVue®	ceVUS	VUR	N/A
2015, Faizah (32)	SonoVue®	ceVUS	VUR	No
2014, Papadopoulou (33)	SonoVue®	ceVUS	VUR	(37/1,010) dysuria (*n* = 26, 2.57%), urinary retention (*n* = 2, 0.2%), abdominal pain (*n* = 2, 0.2%), anxiety (*n* = 1, 0.1%) and crying (*n* = 1, 0.1%) during micturition, blood and mucous discharge (*n* = 1, 0.1%), increased frequency of micturition (*n* = 1, 0.1%), vomiting (*n* = 1, 0.1%), perineal irritation (*n* = 1, 0.1%), and an episode of urinary tract infection 10 days after ce-VUS (*n* = 1, 0.1%).
2014, Wong (34)	SonoVue®	ceVUS	VUR	No
2014, Woźniak (35)	SonoVue®	ceVUS	VUR	N/A
2013, Woźniak (36)	SonoVue®	ceVUS	VUR	N/A
2012, Duran (37)	SonoVue®, Levovist®	ceVUS	VUR, congenital stenosis of the bulbar urethra, diverticulum of the prostatic utricle, PUV	No
2012, Ključevšek (38)	SonoVue®	ceVUS	VUR	No
2010, Kis (39)	SonoVue®	ceVUS	VUR	No
2009, Duran (40)	Levovist®	ceVUS	Posterior urethral valve, diverticulum of prostatic utricle, anterior urethral stricture, intravesical ureterocele	N/A
2009, Kopac (41)	Levovist®	ceVUS, ceGS	Urogenital sinus	N/A
2009, Papadopoulou (42)	SonoVue®	ceVUS	VUR	No
2007, Giordano (43)	Levovist®, SonoVue®	ceVUS	VUR	N/A
2006, Papadopoulou (44)	Levovist®	ceVUS	VUR	No
2005, Berrocal (45)	Levovist®	ceVUS	VUR, PUV, stenosis of the anterior urethra	N/A
2005, Darge (46)	Levovist®	ceVUS	VUR	N/A
2004, Ascenti (47)	SonoVue®	ceVUS	VUR	No
2004, Vassiou (48)	Levovist®	ceVUS	VUR	No
2004, Xhepa (49)	Echovist®, Levovist®	ceVUS	VUR, PUV	No
2003, Ascenti (50)	Levovist®	ceVUS	VUR	N/A
2003, Darge (51)	Levovist®	ceVUS	VUR, IRR	
2003, Maté (52)	Levograf	ceVUS	PUV	N/A
2003, Uhl (53)	Levovist®	ceVUS	VUR	Slight dysuria
2002, Darge (54)	Levovist®	ceVUS	VUR	N/A
2001, Darge (55)	Levovist®	ceVUS	VUR	N/A
2001, Escape (56)	Levovist®	ceVUS	VUR	N/A
2001, Valentini (57)	Levovist®	ceVUS	VUR	No
1999, Mentzel (58)	Levovist®	ceVUS	VUR	No
1998, Bosio (59)	Echovist®, Levovist®	ceVUS, ceRUG	Primary VUR, secondary VUR (hypospadias, duplex kidneys with or without a ureterocele, Hutch diverticulum)	No
1994, Kaneko (60)	Albunex®	ceVUS	VUR	No

ceVUS, contrast-enhanced voiding urosonography; ceRUG, contrast-enhanced retrograde urethrosonography; ceGS, contrast-enhanced genitosonography; UCA, ultrasound contrast agent; VUR, vesicoureteral reflux; IRR, intrarenal reflux; N/A, not available; PUV, posterior urethral valves.

We also searched 24 related narrative and systematic reviews, of which 20 (%) articles involved ceVUS in diagnosing VUR and 4 involved ceRUG and ceGS for other lower genitourinary anomalies (Table 2). When reviewing these clinical trials and reviews, we could get some information on the evolution history, dosages, and safety of ceVUS, ceRUG, and ceGS in pediatrics simultaneously. Hence, we also described the related content referring to the evolution history, dosages, and safety of ceVUS, ceRUG, and ceGS in this review.

**Table 2:** Included reviews of intracavitary contrast-enhanced ultrasonography including ceVUS, ceRUG, and ceGS in pediatric lower genitourinary anomalies performed with UCAs published in English.

Publication year, first author	Intracavitary CEUS technique	Urinary abnormalities
2021, Barnewolt (61)	ceVUS, ceRUG	Urethrovaginal reflux, spinning top urethra, periurethral cysts or abscesses, Gartner duct cysts, PUV, AUV, prostatic utricle, syringocele, urethral duplication, urethral strictures, ureterocele, ectopic ureter, urethral diverticula
2021, Chow (62)	ceGS	Urogenital sinus
2021, Didier (63)	3D/4D ceVUS	VUR
2021, Ntoulia (64)	ceVUS	VUR, IRR, peri-ureteral diverticulum, intravesical ureterocele
2021, Sofia (65)	ceVUS	VUR
2020, Barr (66)	ceVUS	VUR
2020, Viteri (67)	ceVUS	VUR
2019, Chua (68)	ceVUS	VUR
2018, Ntoulia (69)	ceVUS	VUR
2018, Yusuf (70)	ceVUS	VUR
2017, Duran (71)	ceVUS	VUR, megaureter, ectopic ureter, ureterocele, diverticulum, urogenital sinus PUV, AUV, diverticula of the prostatic utricle, stricture of the bulbar urethra, ventral urethral ectasia, spinning top urethra
2017, Sidhu (72)	ceVUS	VUR
2014, Stenzel (73)	ceVUS	VUR
2014, Schreiber-Dietrich (74)	ceVUS	VUR
2014, Tse (75)	ceVUS	VUR
2013, Darge (6)	ceVUS	VUR
2013, Ignee (76)	ceVUS	VUR
2012, Dietrich (77)	ceVUS	VUR
2011, Darge (78)	ceVUS	VUR
2011, McCarville (79)	ceVUS	VUR
2007, Zimbaro (80)	ceVUS	VUR
2004, Darge (81)	ceVUS	VUR
2002, Darge (82)	ceVUS	VUR
2002, Riccabona (83)	ceVUS	VUR

ceVUS, contrast-enhanced voiding urosonography; ceRUG, contrast-enhanced retrograde urethrosonography; ceGS, contrast-enhanced genitosonography; UCA, ultrasound contrast agent; PUV, posterior urethral valve; AUV, anterior urethral valve; 3D/4D, three-dimensional/four-dimensional; VUR, vesicoureteral reflux; IRR, intrarenal reflux.

## The evolution of ceVUS, ceRUG, and ceGS in pediatrics

In 1976, VUR was first diagnosed with ultrasound using M-mode imaging by detecting the changes in renal pelvis echoes in three adults (84). Since then, varieties of attempts including using indirect or direct ultrasound methods in the diagnosis of VUR have been implemented. Due to low sensitivity and specificity to reliably predict VUR, indirect methods without instilling any kind of material into the bladder using B-mode or duplex and color Doppler ultrasound were abandoned (85, 86). Direct ultrasound methods with the administration of various substances (e.g., saline, iodine, air bubbles made by shaking saline or iodine, or adding carbon dioxide, or pure air) into the bladder appeared in the 1980s (87–90). However, the direct ultrasound methods mentioned above have many limitations. One is a false-positive with saline caused by transient dilatation of the renal pelvis due to bladder filling or pre-existing ureteral and/or pelvicalyceal dilatation. The other is a false-negative due to an acoustic shadow caused by air preventing or obscuring reflux of air into the terminal ureters and possible pelvicalyceal dilatation. Another is that the air bubbles lack homogeneity and quantity, and dissolve fast (20–30 s), limiting the time available to detect reflux. Later in 1994, it was first reported that a novel UCA consisting of sonicated air-filled human albumin microspheres called Albunex® was applied to diagnose VUR in a child (60). In 1995, von Rohden et al. used Echovist® (an air-filled microbubble with a galactose shell) to examine VUR (91). However, both Albunex® and Echovist® did not last a long time (approximately 5 min), which is impractical for sufficient reflux evaluation.

The breakthrough in the ultrasound diagnosis of pediatric VUR came in the mid-1990s as the first-generation UCA, Levovist® (air-filled microbubbles with galactose and palmitic acid), became available for clinical practice in Europe (92). It was stable and the only approved UCA for intravesical use in children in 13 European countries and in Australia, but is no longer marketed currently. In the early 2000s, the second-generation UCAs, Lumason®/SonoVue® (sulfur hexafluoride lipid-type A microspheres; Lumason® in the United States and SonoVue® outside the United States) and Optison® (perflutren protein-type A microspheres), came into use. As the second-generation UCAs are even more stable, have fewer adverse events, and need less dose to achieve comparable contrast enhancement compared with Levovist®, it is recommended to use the second-generation UCAs in performing ceVUS.

The technique with the introduction of UCAs into the bladder was widely known as ceVUS since 2000. Before that, different names about this technique were used, such as cystosonography, contrast sonography, micturition sonography, ultrasound cystography, echo-enhanced cystosonography, and retrograde echocystography (59, 89, 93–96). But the use of ceVUS in pediatrics was not approved by the official organization until 22 December 2016; the US Food and Drug Administration (FDA) approved intravesical administration of Lumason® for pediatric suspected or known VUR under the collaboration of both the Society for Pediatric Radiology (SPR) CEUS task force and the International Contrast Ultrasound Society (ICUS). Encouragingly as a milestone event, the FDA's approval of ceVUS in pediatrics stimulated more scholars to get into this sort of research for children, thus promoting the development of intracavitary CEUS applications in VUR and other genitourinary system anomalies. In June 2017, the European Medicines Agency approved the use of SonoVue® for ceVUS in pediatrics. In China, SonoVue® was also approved by the Chinese National Medical Products Administration for the intravesical use in VUR. It is worth mentioning that ceVUS is the only officially approved intracavitary CEUS indication in children.

In addition to the most common application in VUR, ceVUS can also be used to identify urethral abnormalities both in girls and boys. It can be dated back early to the 1980s when several authors used non-voiding ultrasound to diagnose PUV as the dilated posterior urethra could be easily detected by gray-scale ultrasonography (97, 98). Although non-distended urethra can be visualized on B-mode ultrasound, distending the urethra during physiological voiding or with retrograde administration of saline into the bladder or urethra is preferable (45, 99). Recently, with the introduction of UCAs, the urethral sonographic images have been improved significantly. As the length of the female urethra is short, the voiding phase as a natural extension of ceVUS is enough to detect urethral structural and functional abnormalities associated with voiding disorders. While in males, detection of the urethra involves anterior and posterior urethra mainly associated with obstructing pathologies, including PUV, AUV and diverticula, and urethral strictures. To achieve high-resolution ultrasound images of the anterior urethra, ceRUG through retrograde instillation of contrast agents is used primarily for evaluating urethral strictures.

Another application of intracavitary CEUS in children is ceGS. It is a novel technique of instilling UCAs into the perineal orifice to reveal the connections between the urethra and müllerian duct remnants (vagina and prostatic utricle) mainly for diagnosing the urogenital sinus or cloacal malformation. The ceGS technique could be performed as a standalone imaging technique or as an extension of ceVUS. Kopac et al. first reported in 2009 that ceGS combined with ceVUS using Levovist® as a contrast agent was applied to identify urogenital sinus in a female newborn baby admitted due to the the absence of a normal vaginal opening (41). Since then, only a small quantity of specialized pediatric centers performed ceGS in a few cases (7, 24). Although normal saline can also be instilled into the perineal orifice to delineate the urogenital anatomic structure, the administration of UCAs makes revealing the anatomical connections more conspicuous.

## Appropriate dosages and safety of UCAs in pediatric ceVUS, ceRUG, and ceGS

There are two ways of performing ceVUS with the introduction of UCAs into the bladder. The first one is injecting UCAs directly into the bladder. Through this technique, SonoVue®/Lumason® was reported to be administered at a dose of 0.5 ml–2.5 ml, among which most authors preferred a dose of 0.5 ml or 1 ml (18, 31, 32, 100). The other method is infusing UCA/normal saline solution into the bladder. When using the second method, the concentration of the bladder filling volume ranges from 0.2% to 1% with SonoVue®/Lumason® and 0.06%–0.5% with Optison® (9, 28). However, no matter which method is selected, the current recommended dose of SonoVue®/Lumason® is 1 ml. Moreover, with recent great improvements of ultrasound scanner technologies, the ability of contrast-specific imaging modality to clearly depict the microbubbles increased, and it might be too high for a 1 ml dose.

For scanning the urethra through either the voiding or the retrograde method, the dose of UCAs is similar to that used for detecting VUR (i.e., 0.1%–0.2% of UCA/normal saline solution). However, when the retrograde technique is performed, it might require a higher dose of UCAs due to the use of higher-frequency linear probes and the superficial site of the examined region.

The dosage and concentration of UCA/normal saline dilution that is used for ceVUS can also be used equally for ceGS but is variable due to the different types of UCAs and the sensitivity of the ultrasonic device and software. In the limited number of case reports or several case series about ceGS studies, a 5% of bladder filling volume with Levovist®, a 0.1% solution of Lumason®, or a 0.06% Optison®/normal saline solution was used (7, 24, 41).

In general, the ceVUS technique is quite safe. A review of 31 studies encompassing 12,362 children who underwent ceVUS examinations using the second-generation UCAs SonoVue®/Lumason® or Optison® concluded that there were no serious adverse events (101). Among the 31 studies, only two reported 38 non-serious adverse events (0.31%) including dysuria (*n* = 27), transient abdominal pain or discomfort (*n* = 2), urinary retention (*n* = 2), anxiety during voiding (*n* = 1), crying during voiding (*n* = 1), frequency of urination (*n* = 1), blood and mucous discharge (*n* = 1), vomiting (*n* = 1), perineal irritation and discomfort (*n* = 1), and urinary tract infection (UTI) (*n* = 1). All these non-serious adverse events present subacute onset and are self-limited, with uneventful outcomes. They are more likely attributed to the catheterization into the bladder leading to discomfort or its psychological impact on the children than the UCA itself. A relatively small number of studies performed with ceGS reported no adverse events (24).

## Clinical applications of intracavitary CEUS in lower genitourinary anomalies

### Vesicoureteral reflux

**Figure 2:**
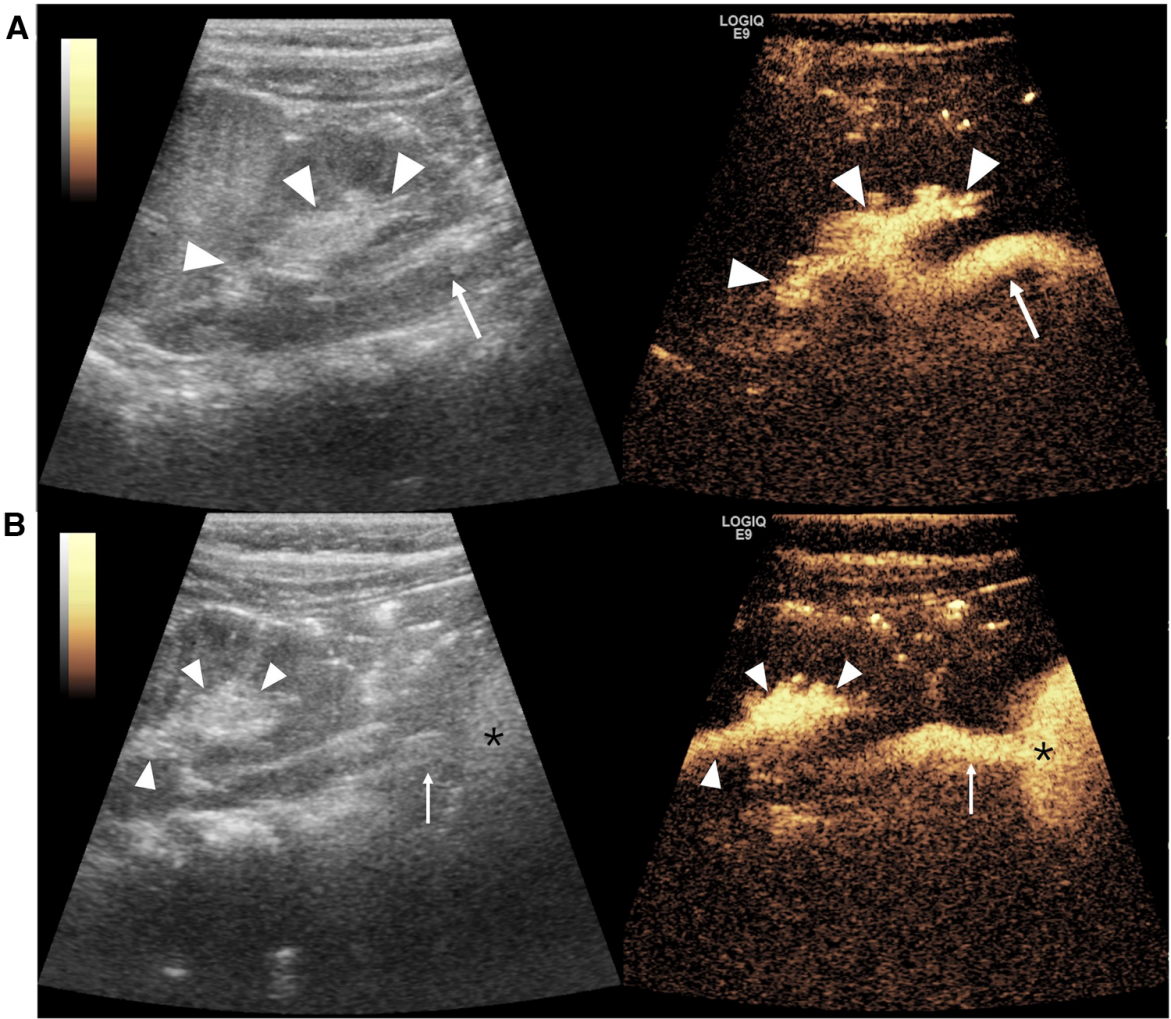
Vesicoureteral reflux imaging on contrast-enhanced voiding urosonography of the dual-screen mode of the left kidney in a 1-year-old boy. (**A**) The sagittal plane of the left kidney displays contrast agent refluxing into the ureter (arrow) and pelvicalyceal system (arrowheads), indicating grade III reflux. (**B**) Panoramic coronal view image: simultaneous visualization of the left kidney (arrowheads), ureter (arrow), and bladder (asterisk) from the left flank full of echogenic microbubbles in a single view.

**Figure 3:**
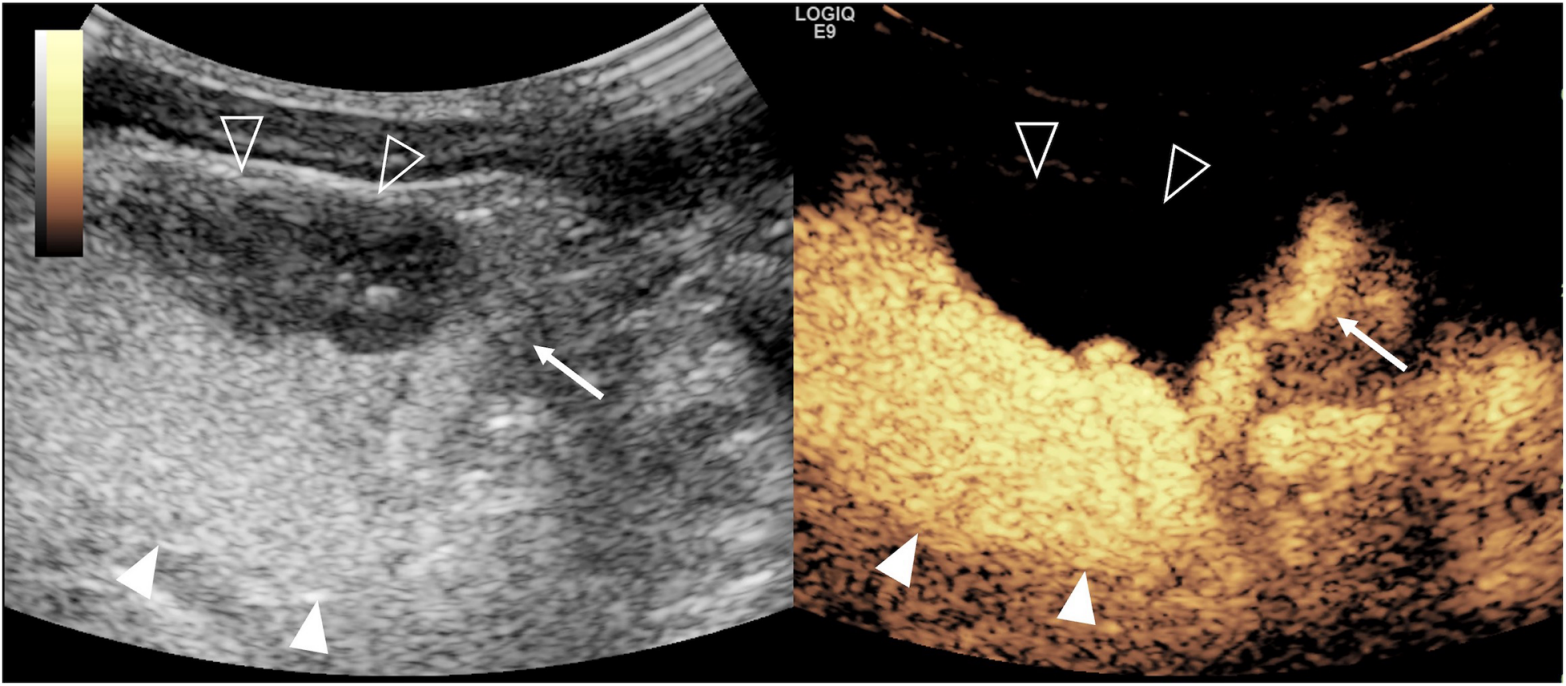
Duplex pelvis complicated with vesicoureteral reflux on contrast-enhanced voiding urosonography (ceVUS) of the dual-screen mode of the left kidney in a 14-day-old girl. Sagittal gray-scale ultrasound (left) and ceVUS (right) images show hydronephrosis of the upper moiety (solid arrowhead) of the duplex kidney and microbubbles refluxing and filling the upper moiety pelvicalyceal (solid arrowheads) with marked dilation and papillary impressions invisible and tortuous dilated ureter (arrow), indicating grade V reflux. There is no dilation and reflux of the lower moiety pelvicalyceal system (open arrowheads).

VUR is the most common urinary tract anomaly in children with a high prevalence of 31.1% in children with UTI (102). It is defined as the presence of UCA microbubbles in the ureter and/or pelvicalyceal system (Figure 2). VUR can be solitary or complicated with other congenital anomalies (e.g., PUV, duplex pelvis, crossed fused renal ectopia, ureteropelvic junction stenosis, prune belly syndrome, and neurogenic bladder) (Figure 3). Solitary VUR is also called primary VUR, which is present at birth due to the deficiency in the ureterovesical junction impairing the anti-reflux mechanism. When VUR is secondary to neurogenic bladder, video urodynamic studies should be recommended as the gold standard to evaluate the function of the lower urinary tract early and to regularly follow up and monitor the deterioration of bladder function for the first few years (103).

**Figure 4:**
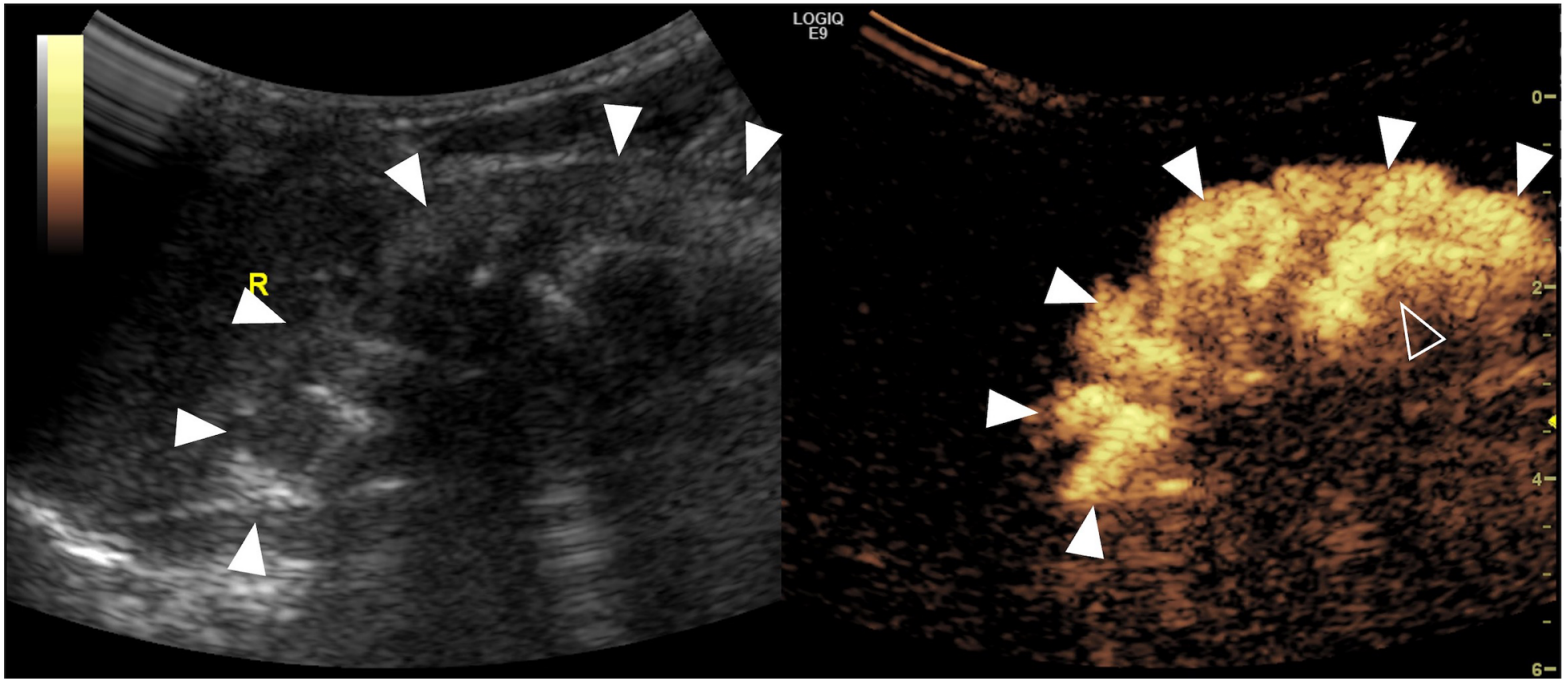
Severe intrarenal reflux complicated with vesicoureteral reflux on contrast-enhanced voiding urosonography of the dual-screen mode of the right kidney in a 14-day-old girl. Sagittal images of the right kidney show microbubbles refluxing into the pelvicalyceal system (open arrowheads) and diffusing into the entire renal parenchyma (solid arrowheads).

Intrarenal reflux occurs in 3%–10% of VUR patients, indicating an increased risk of renal scar formation especially due to high-pressure and high-grade reflux and acting as a risk factor for reflux nephropathy (Figure 4). A VUR-associated reflux nephropathy can lead to chronic kidney insufficiency and end-stage renal failure. So early diagnosis of VUR accurately, especially high-grade VUR, is crucial for patient outcomes. Traditionally, VCUG is recommended as the gold standard for diagnosing VUR in children. However, ceVUS with no radiation is gradually becoming an alternative imaging technique to VCUG as it is not only comparable or even superior compared with VCUG in the sensitivity of detecting VUR but also can detect higher grades of VUR (9). A meta-analysis published in 2019 revealed that the pooled AUC, sensitivity, and specificity of the second-generation UCA with harmonic imaging in diagnosing VUR compared with VCUG for pediatrics were 0.965, 90.43%, and 92.82%, respectively (104). In addition, no matter whether the first-generation or second-generation UCAs are used, the diagnostic value of ceVUS in diagnosing VUR is very high (105).

However, there are still 3% of false-negative rates, which is a limitation (105). False-negatives occur when ceVUS is performed after other diagnostic procedures, such as intravenous injection of iodine or gadolinium contrast media, or intravesical injection of iodine because of higher specific gravity of the residual contrast material depositing at the bottom of the bladder preventing the reflux of the UCA into the ureters. But this is a rare situation that can be avoided by performing the ceVUS examination on different days. Another reason is that the residual urine within the bladder does not optimally mix with the UCAs due to incomplete emptying of the bladder after bladder catheterization. To avoid this situation, ceVUS examinations should always be started with an empty or urine-free bladder.

### Megaureter

A megaureter is defined as a markedly dilated ureter (larger than 7 mm in diameter) regardless of the specific pathological conditions (106). In 1976, the International Conference on Pediatric Urology classified megaureter into three categories: obstructed, refluxing, and non-refluxing non-obstructed, each of which could be subdivided into primary and secondary megaureter according to its pathogenesis. Later in 1980, King noted the presence of distal obstruction in refluxing megaureter and added the category of refluxing megaureter with obstruction (Table 3) (107). Especially when megaureter is combined with VUR, contrast material refluxing into the distal dilated ureter, even the pyelocalyceal system, could be detected by ceVUS.

**Table 3:** The classification of megaureter.

Classification	Primary	Secondary
Non-refluxing and non-obstructed	Most common in neonates and idiopathic (immature development and function of VUJ)	Urinary tract infection and diabetes insipidus
Refluxing	Dysfunction of the VUJ	Bladder outlet obstruction, neurogenic bladder, and other bladder dysfunctions
Obstructed	Functional obstruction or anatomic stricture of the distal ureter	Infra-vesical obstruction (neurogenic bladder, ureterocele, posterior urethral valve, etc)
Refluxing and obstructed	No peristalsis of the distal ureter and ureteral reflux	Ectopic ureter inserting into bladder neck regurgitates as the bladder relaxes and obstructs as it tights

VUJ, vesicoureteric junction.

### Ectopic ureter

An ectopic ureter is described as the ureter inserting into the abnormal location except for the trigone. Complete duplex kidney is the most common phenotype of duplex collecting systems, which has two separate ureters called as the upper and lower moiety ureters. An ectopic ureter is usually associated with the upper moiety ureter based on Weigert–Meyer rule. The ectopic ureter opens into the proximal urethra, seminal vesicles, vas deferens, or ejaculatory track in boys; the distal urethra, vagina, or uterus in girls; and the bladder neck both in boys and girls. VUR can happen both in the upper and lower moieties with the lower moiety the frequent location (108), which can be detected by ceVUS. It is also possible for ceVUS to detect the openings of the ectopic ureters.

### Ureterocele

Ureteroceles are focal cystic dilations of the intravesical portion of the distal ureter caused by congenital obstruction and may exist solitary or associated with a complete ureteral duplication. When occurring in the latter circumstance, they are associated with the ectopic insertion of the upper moiety ureter (109). According to the Glassberg classification system, ureteroceles are classified in two categories: the orthotopic type and the ectopic type. In the former case, ureteroceles are entirely located intravesical on the trigone and usually associated with the single system, while in the latter case, ureteroceles are extravesical or ectopic with a portion situated near the bladder neck or urethra and associated with the complete duplication. Ureterocele-associated pathophysiologic features (i.e., upper moiety obstruction caused by intravesical ureteral obstruction and VUR to the ipsilateral lower moiety ureter) can be perceived by ceVUS.

### Bladder diverticulum

**Figure 5:**
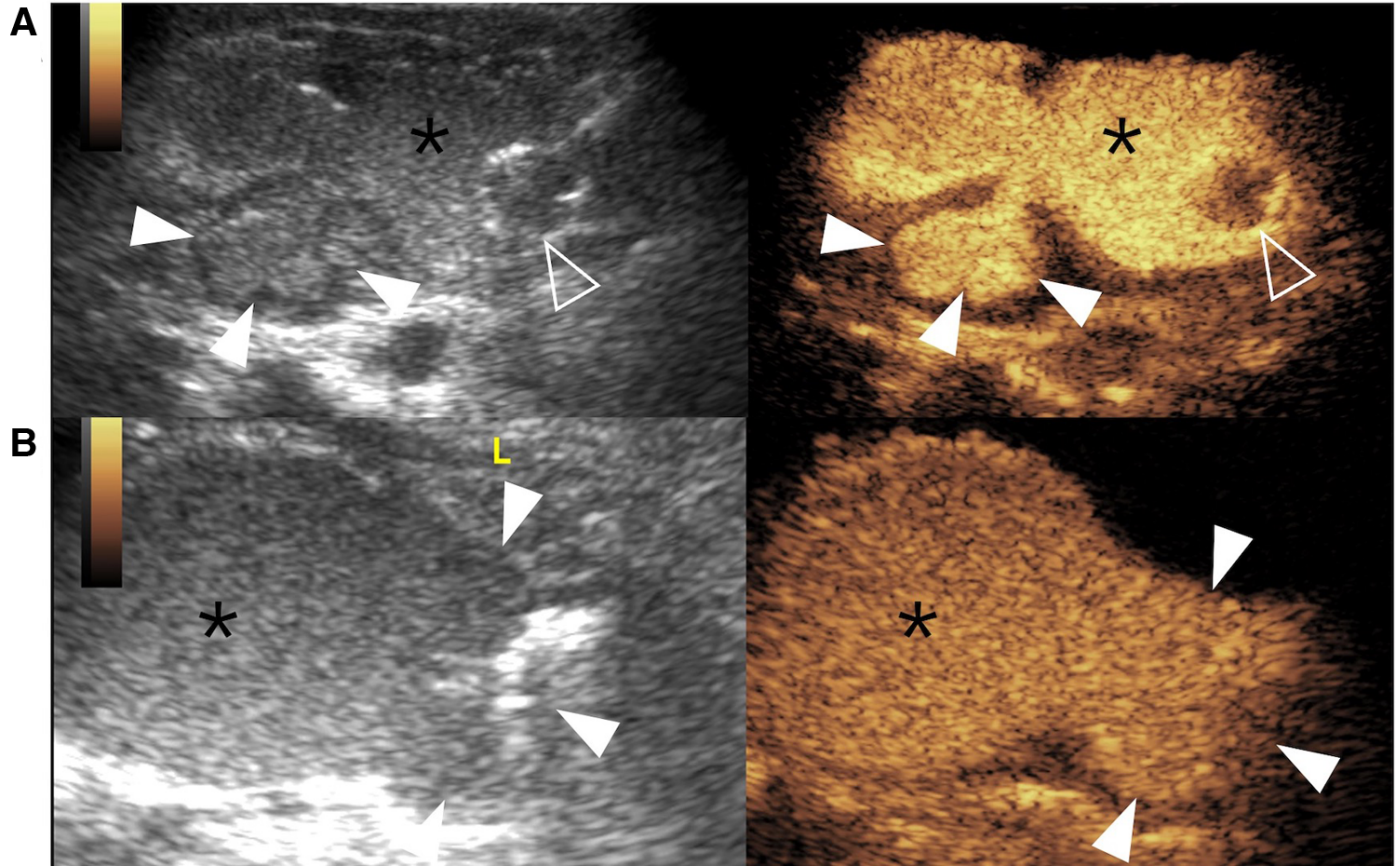
Bladder diverticulum on contrast-enhanced voiding urosonography of the dual-screen mode in a 3-year-old boy (**A**) and a 1-year-old boy (**B**). (**A**) The sagittal plane of the bladder (asterisks) shows a contrast-filled saccular-like diverticulum (solid arrowheads) behind the bladder. The open arrowhead indicates the echogenic balloon. (**B**) The transverse plane of the bladder (asterisks) shows a large contrast-filled diverticulum (solid arrowheads) on the left of the bladder.

Bladder diverticulum is rare in the pediatric populations, described as a protrusion of the bladder mucosa and submucosa due to the weakness of the muscularis propria in the detrusor musculature of the bladder wall, resulting in poor emptying function (Figure 5). It can be congenital or acquired. The similarities of both types are that they have the same pathologies and histological examinations of “hypoplasia of the muscularis layer”.

Congenital or primary diverticulum occurs infrequently and can be usually single and unilateral. It is usually present during childhood with normal intravesical pressures in the absence of a bladder outlet obstruction and mainly caused by congenital connective tissue diseases, such as Ehlers–Danlos syndrome, Williams–Beuren syndrome, or Menkes kinky hair syndrome (110, 111). On cystoscopy, it reveals a smooth wall and is usually seen next to the vesicoureteric junction. Acquired or secondary diverticula are more common than the congenital types, usually multiple and resulting from any part of the bladder wall. On cystoscopy, they display multiple bladder trabecular changes. This type can be a result of high intravesical pressure caused by bladder outlet obstruction or detrusor-sphincter dyssynergia during micturition with the typical representative of neurogenic bladder.

Hutch diverticulum, also called as periureteral diverticulum, is a rare special type of congenital diverticula, located posterior-lateral to the ureteral orifice apart from the trigone, and developing to incorporate it from a small herniation (112). Ipsilateral long-standing VUR is one of the most common complications associated with it, and ceVUS is a valuable method to demonstrate VUR.

### Posterior urethral valves

PUVs, only occurring in males, are the most common urethral obstructed anomaly and have high risks to result in end-stage renal failure. Its incidence is approximately 1/5,000–8,000. It is well known that PUVs are classified into three types according to the anatomic structure characteristics of the obstructed location firstly proposed by Young et al. (113) in 1919 and later precisely explained by Douglas Stephens in 1996. Clinically, type 1 and type 2 are the two common main types. Thereinto, type 2 is considered to be a normal anatomic variant and seems to be overestimated. Type 1 has a valvular shape oblique to the urethral axis, originating in abnormally located Wolffian duct orifices. Its verumontanum is larger than normal and continuous to the lesion, with the inferior crest thick like a fin. Type 3 has a membrane or diaphragm transverse to the urethral axis with a central hole, occurring from a persistent urogenital membrane. It has a relatively small verumontanum, which is discontinuous to the lesion, and the inferior is very thin. Early diagnosis, accurate discrimination of type 1 from type 3, and prompt treatment can prevent the progress of urethral obstructions and are helpful for improvements of future prognosis.

The findings of PUVs at ceVUS demonstrate bladder thickening and irregularity with diverticula formation, dilatation of the posterior urethra with a diameter of at least 7 mm, lumen narrowing of the valve area, decreased caliber of the anterior urethra, and difficulties of the contrast agents getting through the valve region (99). PUV is one common cause resulting in VUR. Some authors can also diagnose PUVs on B-mode ultrasonography (114).

### Anterior urethral valves

AUVs are rarer and less frequent than PUVs in congenital lower urinary obstruction but are the most common cause of anterior urethral obstruction. Similar to PUV, AUV can also lead to renal failure. The valvular mucosa folds of AUV arise from the ventral part of the urethra and uprise during voiding, causing obstruction of the urine flow. The shape of the valves can be mostly semilunar (70%) and fractionally iris-like (30%). The location of AUV is variable, from the most common location the bulbar urethra (40%) to the distal portion of the urethra (e.g., fossa navicularis) (115). By and large, AUVs are usually located near the bulbar urethra or the penoscrotal junction. Clinical manifestations vary depending on visiting ages, obstruction severity, and related complications; presentations mainly include infectious-related symptoms primarily in the newborn and infant and voiding-related symptoms chiefly in the elderly child. VUR, one of the various complications, is highly predictive of poor outcomes, including renal failure and/or death. Sometimes longitudinal trans-penile B-mode ultrasonography can be used to diagnose AUVs during micturition. It is equivocal between the relationship of AUV and the anterior urethral diverticula as they often occur simultaneously (33%) (116). Normally, the discrepancy between the posterior and anterior urethral distention ranges from 0 to 2 mm. But in patients with AUV, we can observe the dilatation of the proximal urethra, the valve structure shown as a linear filling defect, and the prominent narrowing of the distal urethra.

### Diverticula of the prostatic utricle

The normal prostatic utricle is a small, blind-opening pouch located in the midline of the verumontanum (i.e., the floor of the prostatic urethra), with columnar or cuboidal epithelium lining, communicating with the urethra. When enlarged to at least 1 cm, it is considered pathological, which is lined by squamous epithelium. The most common symptoms of diverticula of the prostatic utricle include UTI, postvoid dribbling, and epididymitis. Enlarged prostatic utricles are frequently seen in boys with intersex, prune belly syndrome, Down syndrome, cryptorchidism, or hypospadias (11%–14%) (117, 118).

### Stricture of the bulbar urethra

The causes leading to stricture of the bulbar urethra can be inflammatory, traumatic, or congenital. While most urethral bulbar strictures are the result of infectious urethritis, straddle injury, or other iatrogenic instrumentation causes, congenital stricture of the bulbar urethra is rare and referred to as obstructive Cobb's collar, Moormann's ring, or Young's type III valve, which is caused by disabling of canalization of the cloacal membrane in the development of fetuses. The ceVUS technique can not only assess the length and thickness of bulbar urethral stricture but also differentiate the congenital stricture of the bulbar urethra from segmental strictures, which is important for surgery planning. Congenital stricture of the bulbar urethra at ceVUS demonstrates a focal narrowing of the bulbar urethra, a dilated urethra proximal to the stricture, and a normal penile urethra. The site of narrowing in the bulbous urethra is more distal to the external urethral sphincter, which is different from PUV. Patients with bulbar urethral stricture present with UTI or diurnal enuresis, and VUR takes up to 53% of the cases (119).

### Spinning top urethra

Spinning top urethras are named for the markedly dilatated posterior urethra presenting a fusiform distal-end appearance and mainly in females. It is used to be considered a normal variant, but recently, some investigators have proposed that these conditions should hint at the lower urinary tract functional disorders. Spinning top urethra can be associated with bladder disturbance, recurrent UTI, and voiding dysfunction, as well as VUR. As ceVUS is a real-time tool, it can identify spinning top urethra, which is only present during the early voiding phase and resolves in the later voiding phase.

### Urogenital sinus

Persistent urogenital sinus (PUGS) is one form of congenital developmental cloacal anomalies, of which the incidence is about 6/100,000 (120) and only occurs in females. Its anatomical characteristic is described as a single common channel where the urethra and vagina converge due to the failure of urethra–vaginal septum formation, while the normal orifice of the anus exists. It can be isolated or has an association with a variety of diseases including congenital adrenal hyperplasia, McKusick–Kaufman syndrome, or Bardet–Biedl syndrome. There are two types of PUGS according to the length of the common PUGS: high (>3 cm) and low (<3 cm). Accurately distinguishing from the two types and determining the level where the urethra opens are critically crucial for surgery planning strategies. The technique of ceGS combined with ceVUS can delineate the anatomical abnormalities of PUGS, reveal the common passage of urethra and vagina, and measure the length of urethra and the common route (121). As it is sometimes challenging to evaluate the anatomy of the urogenital sinus, a three-dimensional or four-dimensional ultrasound reconstruction and respective post-processing techniques could be used to improve the diagnostic accuracy (24). CeGS alone is also used to evaluate the effect of surgical treatment and follow up for complications after surgery (62).

## Discussion

In this narrative review, we elaborated on the evolution history of intracavitary CEUS techniques (ceVUS, ceRUG, and ceGS) and further introduced their appropriate dosages and safety in pediatrics. Most important of all, we described related pediatric clinical applications of ceVUS, ceRUG, and ceGS in lower genitourinary diseases (e.g., VUR, megaureter, ectopic ureter, ureterocele, bladder diverticulum, PUV, AUV, diverticula of the prostatic utricle, stricture of the bulbar urethra, spinning top urethra, and urogenital sinus).

Most of the published studies and reviews were related to ceVUS in evaluating VUR (64). Only a few case reports or case series and reviews were about other genitourinary diseases (7, 24, 62). Compared with previous reviews, ours is the first comprehensive and systematic review to describe the evolution history, appropriate dosages and safety of intracavitary CEUS techniques (ceVUS, ceRUG, and ceGS), and their clinical applications in lower genitourinary diseases in pediatrics.

As the only officially approved intracavitary CEUS application in children worldwide, ceVUS has many advantages compared with fluoroscopy because it is safe, radiation-free, real-time, and bedside or intraoperatively performed (6, 122). As numerous evidence-based medical studies have shown that ceVUS is comparable with or even more sensitive in detecting VUR than VCUG or RNC in routine diagnostic scenarios, it has much potential to complement or even replace traditional radiated procedures in several conditions (107).

Although ceVUS has so many advantages, it is crucial to consider its limitations. Firstly, there is no single uniform standardization to perform this approach requiring individual case-by-case examination. In addition, some inherent limitations regarding ultrasound use should be taken into consideration (e.g., operator dependence, bowel gas interference, and difficulties obtaining some anatomical regions). Finally, the biggest limitation of ceVUS is that it cannot present panoramic views leading to the incapability of visualizing the urethra and reno-ureteral system at one view during voiding (80). Sometimes, to assess the urethra simultaneously, VCUG is reserved for evaluating some complex anatomic settings pre-surgically.

Despite intracavitary CEUS ultrasound examinations being off-label except for ceVUS, the European Society of Pediatric Radiology (ESPR) abdominal task force still recommends ceRUG in evaluating the urethra or ceGS in imaging urogenital sinus malformations as an alternative option (24, 123). As a non-irradiating, safe, and sedation-free imaging method, some promising experience of ceRUG and ceGS has been accumulated in appropriate clinical circumstances. The greatest benefit of the two techniques over fluoroscopy is their ability to not only reveal the opacified organs and abnormal connections but also highlight the structures around the organs. However, studies about the applications of ceRUG and ceGS are only limited to a few case reports or small cumulative cohorts. To further explore the value of ceRUG and ceGS in pediatric lower genitourinary anomalies, more excellent studies about ceRUG and ceGS in the future are anticipated.

## Conclusion

We presented three intracavitary CEUS methods including ceVUS, ceRUG, and ceGS in pediatric lower genitourinary anomalies, which have the advantages of no radiation exposure and displaying both morphological and functional imaging. As a safe problem-solving and increasingly welcomed imaging modality, ceVUS is practical and effective and can be a feasible imaging alternative to fluoroscopy because of its high diagnostic accuracy for detecting and grading VUR in pediatrics. In addition, in the evaluation of urethral pathologies and urogenital sinus, ceRUG and ceGS have become promising techniques for their feasibility, efficacy, and no need for sedation. With the advent of 3D printing, we look forward to further significant developments of the intracavitary CEUS technique.
